# Pressure Control of Centrifugal Fan Using Softsign-PI Controller Tuned by Hybrid Starfish Optimization Algorithm with Differential Evolution

**DOI:** 10.3390/biomimetics11050331

**Published:** 2026-05-09

**Authors:** Cebrail Turkeri, Serdar Ekinci, Davut Izci, Dacheng Li, Erdal Akin

**Affiliations:** 1 Department of Computer Engineering, Batman University, 72100 Batman, Türkiye; cebrail.turkeri@batman.edu.tr; 2 Department of Computer Engineering, Bitlis Eren University, 13100 Bitlis, Türkiye; sekinci@beu.edu.tr; 3 Department of Electrical and Electronic Engineering, Bursa Uludag University, 16059 Bursa, Türkiye; 4 Birmingham Centre for Energy Storage, School of Chemical Engineering, University of Birmingham, Edgbaston, Birmingham B15 2TT, UK; d.li.6@bham.ac.uk; 5 Department of Computer Science and Media Technology, Malmö University, 205 06 Malmö, Sweden; 6 Sustainable Digitalisation Research Centre (SDRC), Malmö University, 205 06 Malmö, Sweden; 7 Biofilms Research Center for Biointerfaces (BRCB), Malmö University, 205 06 Malmö, Sweden

**Keywords:** starfish optimization algorithm, differential evolution, Softsign-PI controller, centrifugal fan

## Abstract

This study addresses pressure regulation in an induction-motor-driven centrifugal fan and introduces two complementary novelties: a Softsign-PI controller that shapes the tracking error via a Softsign nonlinearity before PI regulation and a hybrid starfish optimization with a differential evolution (hSFOA-DE) scheme for automatically tuning the controller parameters. The approach is evaluated on an experimentally validated nonlinear fan–motor model and benchmarked against modern metaheuristics—starfish optimization algorithm (SFOA), animated oat optimization (AOO), electric eel foraging optimization (EEFO), differential evolution (DE), particle swarm optimization (PSO)—as well as classical tunings—Murrill-based 2-DOF PID, Tyreus–Luyben PID and Ziegler–Nichols PI. Statistical summaries and boxplots indicate superior central tendency with reduced run-to-run variability; fitness–evolution curves show faster convergence; and time-domain performance metrics confirm improved transient and steady-state behaviour. Objective function comparisons further show the lowest values of both the Zwe-Lee Gaing (ZLG) and integral of absolute error (IAE), supporting advantages in robustness and tracking accuracy of the proposed approach. These gains reduce overshoot and cumulative error, which can lessen throttling losses and actuator duty in fan/pump service, suggesting potential energy and maintenance benefits.

## 1. Introduction

Centrifugal turbomachinery, including pumps, fans, and compressors, are widely employed in industrial processes, water distribution systems, thermal power plants, oil refineries and heating, and ventilation and air conditioning (HVAC) installations, where they are responsible for ensuring reliable fluid transport, precise pressure regulation, and stable flow delivery [1,2,3]. Since a significant share of industrial electricity consumption is attributable to motor-driven systems, particularly pumps and fans, which account for more than 60% of the energy converted by induction motor drives in industrialized countries, efficient and reliable control of these units is of paramount importance [4]. Maintaining stable pressure is vital for preventing cavitation, avoiding excessive mechanical stress, improving energy efficiency, and ensuring operational safety [5,6]. However, the dynamic coupling between the turbomachinery’s hydraulic and electromechanical dynamics results in nonlinear behaviour and significant parameter sensitivity under varying load conditions, making controller design challenging [7]. These factors demand solutions capable of accurate setpoint tracking, robust disturbance rejection, and adaptability to uncertainties.

Although advanced control strategies can offer high-performance solutions for nonlinear turbomachinery systems, their practical deployment in industrial fan and pump applications is often constrained by implementation complexity, computational burden, and dependence on accurate system models. In contrast, proportional-integral (PI)-based control schemes remain widely preferred in industrial environments because of their simplicity, reliability, and ease of maintenance. Therefore, rather than introducing a more complex control paradigm, this study focuses on enhancing the performance of PI-based control through nonlinear error shaping and advanced optimization.

The present study addresses this need by proposing a hybrid starfish optimization algorithm with differential evolution (hSFOA-DE) to tune a novel two-stage controller composed of a Softsign nonlinear block followed by a PI stage. The Softsign block is placed upstream of the PI controller to exploit its smooth saturation characteristics while preserving the steady-state accuracy of PI control. Based on the literature, Softsign has not yet been applied in PI/PID-based control architectures for nonlinear turbomachinery systems such as pumps or fans.

The centrifugal fan-induction motor model employed in this study is based on an experimentally validated nonlinear fan–motor system [8]. While centrifugal pumps and fans differ in operating regimes and certain parameter values, fans typically emphasize aerodynamic loading, whereas pumps emphasize hydrodynamic loading. Nevertheless, they share the same underlying electromechanical–hydraulic coupling structure. This commonality allows the validated fan model to be generalized to other turbomachinery systems with appropriate parameter adjustments. Using such a validated fan model provides a reliable basis for evaluating the proposed control strategy through reference tracking experiments in the MATLAB/Simulink (2024b) environment.

The proposed hSFOA-DE-tuned Softsign-PI controller is compared against both classical empirical tuning methods, namely Murrill-based 2-DOF PID, Tyreus–Luyben PID, and Ziegler–Nichols PI, and modern metaheuristics, including SFOA, AOO, EEFO, DE, and PSO. The evaluation metrics include the integral of absolute error (IAE), Zwe-Lee Gaing (ZLG)-based performance indicator, convergence characteristics, statistical robustness, and reference tracking performance.

The main contributions of this study can be summarized as follows:

• A novel Softsign-PI control structure is proposed, where a Softsign nonlinear function is employed as a prefilter to enhance transient behaviour while preserving the steady-state accuracy of PI control.
• A hybrid optimization framework based on the starfish optimization algorithm and differential evolution (hSFOA-DE) is developed for effective and robust tuning of controller parameters.
• A comprehensive comparative analysis is performed against both classical tuning methods (Murrill-based 2-DOF PID, Tyreus–Luyben PID, Ziegler–Nichols PI) and state-of-the-art metaheuristic algorithms (SFOA, AOO, EEFO, DE, and PSO).
• The proposed method is validated on a literature-based experimentally verified nonlinear centrifugal fan–induction motor model under nominal operating conditions.

The remainder of this paper is organized as follows: Section 2 presents the related work. Section 3 introduces the SFOA, DE, and their hybridization into the proposed hSFOA-DE method. Section 4 describes the mathematical modelling of the centrifugal fan driven by an induction motor. Section 5 presents the structure of the proposed Softsign-PI controller. Section 6 outlines the objective function formulation and the application of hSFOA-DE for controller tuning. Section 7 provides the results and discussion, including comparative evaluations with classical and advanced methods. Finally, Section 8 concludes the paper and suggests directions for future research.

## 2. Related Work

In recent decades, numerous advanced control strategies have been developed for turbomachinery systems. Model predictive control (MPC) has been successfully applied to active surge suppression in compressors and HVAC system [9,10] and pressure regulation for water distribution [11], offering excellent multivariable handling and constraint management. However, MPC’s computational complexity and reliance on accurate plant models often hinder its use in cost-sensitive or resource-constrained environments [12]. Linear quadratic regulator (LQR) designs have also been applied to HVAC fan and duct systems [13], providing satisfactory results in simplified or moderate nonlinear models, but often showing limitations when faced with strong nonlinearities and environmental disturbances. Adaptive control has been investigated for centrifugal pump [14], offering adaptability but with increased tuning and stability assurance complexity.

Similarly, lead–lag compensators [15], sliding mode control (SMC) [16], and cascaded control structures [17] have been applied in pump–motor and HVAC fan systems to enhance robustness, transient response, and disturbance rejection. However, their implementation often involves additional sensing requirements or introduces design complexity. In parallel, observer-based methods such as extended Kalman filters (EKF) [18] and data-driven approaches such as artificial neural networks (ANNs) [19] have been employed for sensorless estimation of pressure and flow rate in turbomachinery. These methods can reduce instrumentation requirements, but they may also increase computational and algorithmic complexity. Therefore, despite the advances achieved, the adoption of advanced controllers in industry remains limited by cost, model dependence, and maintenance requirements.

In practice, proportional-integral (PI) and proportional-integral-derivative (PID) controllers remain the dominant choice in industrial control [20,21]. Their popularity stems from their structural simplicity, ease of tuning, and ability to provide satisfactory performance across a wide range of operating conditions without extensive modelling. Nevertheless, achieving high performance requires accurate parameter tuning. Widely used empirical tuning rules for PI, PID, and their variants, such as those by Murrill [22], Tyreus–Luyben [23], Ziegler–Nichols [24], and Cohen-Coon [25], have been derived from simplified linear process assumptions. These limitations have motivated the use of heuristic and metaheuristic optimization techniques, which offer greater flexibility in handling nonlinear and time-varying systems and have been widely applied for PI/PID tuning.

Methods such as particle swarm optimization (PSO) [26], differential evolution (DE) [27], and genetic algorithms (GA) [28] have been successfully applied to control design. In the optimization literature, DE is generally recognized for its strong convergence in continuous optimization problems, PSO provides flexible parameter adjustment via its cognitive and social acceleration coefficients, and GA is commonly associated with population diversity and exploration. Nevertheless, these methods may still suffer from drawbacks such as premature convergence, parameter sensitivity, or slow refinement in complex search landscapes. More recent bio-inspired techniques, including the starfish optimization algorithm (SFOA) [29], animated oat optimization (AOO) [30], and electric eel foraging optimization (EEFO) [31], have shown competitive performance in engineering optimization tasks. Although SFOA is a relatively recent optimizer, it was selected as the base algorithm because its original formulation provides a structured exploration–exploitation mechanism inspired by exploration, preying, and regeneration behaviours [29]. The original SFOA study reported evaluations on classical benchmark functions, CEC 2017 and CEC 2022 test suites, and comparisons with a large number of metaheuristic algorithms, indicating its potential for continuous engineering optimization tasks [29]. In the present work, SFOA is not treated as a standalone final solution; rather, it is adopted as a structured search framework and further enhanced with DE-based mutation and crossover operators to improve candidate diversification and local refinement during controller tuning.

Hybrid approaches, which combine the strengths of two or more algorithms, have emerged as a promising way to improve the exploration–exploitation balance. For example, Maiti et al. [32] enhanced the crayfish optimization algorithm by integrating DE’s mutation and crossover strategies, achieving faster convergence and improved solution quality. Similarly, Liu et al. [33] proposed a hybrid PSO-DE algorithm, incorporating DE to help PSO escape stagnation and accelerate convergence, resulting in improved performance on both benchmark and engineering optimization problems. These studies indicate that DE-based hybridization can be effective when the base optimizer requires stronger local refinement or improved convergence stability.

Recent studies have also explored Runge–Kutta-based optimization frameworks for nonlinear system identification problems. For instance, Ali et al. [34] and Khan et al. [35] proposed optimization approaches based on numerical integration schemes, demonstrating improved performance in complex nonlinear modelling tasks. However, these studies mainly focus on system identification rather than controller parameter tuning. In contrast, the present study addresses the tuning of a Softsign-PI controller for fan–motor pressure regulation using a hybrid SFOA-DE framework. Overall, the use of hybrid metaheuristics for pressure control in centrifugal fan/pump-induction motor systems remains limited.

Softsign-based nonlinear functions have also recently attracted attention in control applications due to their smooth saturation behaviour. Such functions can limit control effort without introducing abrupt nonlinearities, thereby improving transient smoothness, actuator safety, and noise robustness [36]. For example, Lyu et al. [37] designed a variable Softsign function within an adaptive EKF algorithm for permanent magnet synchronous motor vector control, achieving improved estimation performance compared with conventional EKF methods. Similarly, Li et al. [38] employed a Softsign-based linear–nonlinear tracking differentiator in a backstepping control scheme for an electro-optical tracking system, improving transient response while reducing parameter complexity. Nevertheless, the existing Softsign-based studies have mainly considered estimation, differentiator, or advanced nonlinear control structures. Its direct use as a nonlinear prefilter in a PI/PID-type controller for turbomachinery pressure control has not been reported.

Accordingly, the present work is positioned at the intersection of industrial PI-based control, Softsign nonlinear error shaping, and hybrid metaheuristic parameter tuning. Unlike advanced controllers that may require complex modelling or implementation, the proposed structure preserves the simplicity of PI control while introducing nonlinear shaping through Softsign and automatic parameter search through hSFOA-DE. This provides a practically oriented control framework for nonlinear fan–motor pressure regulation and establishes the research gap addressed in this study.

## 3. Proposed Algorithm

### 3.1. Starfish Optimization Algorithm

SFOA is a population-based stochastic optimization algorithm inspired by the natural behaviours of starfish, including searching, preying, and regeneration mechanisms [39]. In the SFOA structure, each starfish represents a candidate solution, and its position corresponds to a vector of decision variables in the search space. The algorithm mainly operates through two complementary search phases: exploration and exploitation. The exploration phase is responsible for diversifying the candidate solutions over the feasible search domain, whereas the exploitation phase improves the search process around promising regions by using preying and regeneration-based movements.

Let N denote the number of starfish in the population and D represent the number of design variables. The position of the i-th starfish can be expressed as $X_{i}=\left[X_{i,1},X_{i,2},\ldots ,X_{i,D}\right]$. At the beginning of the optimization process, the initial population is randomly generated within the lower and upper bounds of the design variables as follows:

(1) $$X_{i,j}=l_{j}+r\left({u_{j}-l}_{j}\right),\;\;\;\;\;\;\;i=1,2,\ldots ,N,\;\;\;\;\;\;j=1,2,\ldots ,D$$

where $X_{i,j}$ is the j-th component of the i-th starfish, $l_{j}$ and $u_{j}$ denote the lower and upper limits of the j-th design variable, respectively, and r is a uniformly distributed random number in the interval (0, 1). After the initialization stage, all candidate solutions are evaluated by the objective function, and the corresponding fitness values are stored in a vector given by

(2) $$F={\left[\begin{array}{c} F\left(X_{1}\right)\\ F\left(X_{2}\right)\\ \begin{array}{c} \vdots \\ F\left(X_{N}\right) \end{array} \end{array}\right]}_{N\times 1}$$

where F is the fitness vector and $F\left(X_{i}\right)$ denotes the objective function value of the i-th starfish. Based on this vector, the best candidate solution in the current population is identified and stored as $X_{best}$. In the original SFOA formulation, the exploration and exploitation phases are selected with the same probability through the control parameter $G_{p}=0.5$.

#### 3.1.1. Exploration Phase

The exploration phase of SFOA is designed as a dimension-dependent search mechanism. Unlike conventional vector-based updating schemes, SFOA does not necessarily update all variables simultaneously. Instead, it applies either a five-dimensional search pattern or a unidimensional search pattern depending on the dimension of the optimization problem.

For high-dimensional problems where D>5, five randomly selected dimensions are updated. This mechanism is associated with the five-arm structure of a starfish and allows the algorithm to improve search diversity while reducing unnecessary full-dimensional movements. The updating rule for this case is expressed as

(3) $$Y_{i,p}^{T}=\left\{\begin{array}{c} X_{i,p}^{T}+a_{1}\left(X_{best,p}^{T}-X_{i,p}^{T}\right)\cos\theta ,\;\;\;\;\;r\leq 0.5\\ X_{i,p}^{T}-a_{1}\left(X_{best,p}^{T}-X_{i,p}^{T}\right)\sin\theta ,\;\;\;\;\;r>0.5 \end{array}\right.$$

where $X_{i,p}^{T}$ and $Y_{i,p}^{T}$ denote the current and updated positions of the i-th starfish at iteration T for the selected dimension p, respectively. $X_{best,p}^{T}$ is the corresponding component of the current best solution. In this equation, p represents five randomly selected dimensions from the D-dimensional search space. The coefficient $a_{1}$ is calculated as $a_{1}=\left(2r-1\right)\pi$ while θ is defined as $\theta =\left(\pi /2\right)\left(T/T_{max}\right)$, where $T_{max}$ is the maximum number of iterations. The sine and cosine terms provide two alternative movement tendencies around the current best solution, thereby supporting the exploratory behaviour of the population.

For low-dimensional problems where D≤5, SFOA employs a unidimensional search pattern. In this case, only one selected dimension is updated by using the position information obtained from two randomly chosen starfish. The corresponding updating equation is given as

(4) $$Y_{i,p}^{T}=E_{t}X_{i,p}^{T}+A_{1}\left(X_{k_{1},p}^{T}-X_{i,p}^{T}\right)+A_{2}\left(X_{k_{2},p}^{T}-X_{i,p}^{T}\right)$$

where $X_{k_{1},p}^{T}$ and $X_{k_{2},p}^{T}$ are the p-dimensional components of two randomly selected starfish, while $A_{1}$ and $A_{2}$ are random numbers in the interval $\left(-1,\;1\right)$. The term $E_{t}$ denotes the energy factor and is calculated as $E_{t}=\left(\left(T_{max}-T\right)/T_{max}\right)\cos\theta$. This updating strategy enables the algorithm to perform a focused search in low-dimensional problems while still preserving stochastic movement through the information received from other candidate solutions.

After the exploration update, the feasibility of each generated candidate solution is checked with respect to the lower and upper bounds. If an updated component violates the feasible search range, the previous feasible value is retained according to the original SFOA mechanism.

#### 3.1.2. Exploitation Phase

The exploitation phase of SFOA is constructed using preying and regeneration behaviours. The preying mechanism is the main local search component of the algorithm and uses the relative distance between the current best solution and selected starfish to guide the population toward promising regions. In this mechanism, five distance vectors are first calculated as $d_{m}=X_{best}^{T}-X_{m_{p}}^{T}$, where m=1,2,…,5, and $m_{p}$ denotes five randomly selected starfish from the population. Then, two of these distance vectors are randomly selected and used to update the position of each candidate solution as follows:

(5) $$Y_{i}^{T}=X_{i}^{T}+r_{1}d_{m_{1}}+r_{2}d_{m_{2}}$$

where $r_{1}$ and $r_{2}$ are random numbers in $\left(0,1\right)$, and $d_{m_{1}}$ and $d_{m_{2}}$ are two randomly chosen distance vectors from the calculated distance set. This two-directional search structure allows the candidate solutions to exploit the neighbourhood of the best solution while maintaining a certain degree of stochastic movement.

In addition to the preying mechanism, SFOA includes a regeneration strategy. In the original algorithm, this mechanism is applied only to the last starfish in the population, namely i=N. The regeneration-based update is formulated as:

(6) $$Y_{i}^{T}=exp\left(-\frac{TN}{T_{max}}\right)X_{i}^{T}$$

where T is the current iteration, N is the population size, and$\;T_{max}$ is the maximum number of iterations. The exponential term gradually modifies the movement effect as the search process proceeds. Although the regeneration mechanism is applied to only one candidate solution in each relevant iteration, it provides an additional search behaviour that may help the algorithm reduce premature stagnation.

After the exploitation update, the generated positions are checked against the predefined lower and upper bounds. If any component exceeds the feasible range, it is adjusted to the corresponding boundary value. Finally, the fitness values of the updated starfish are evaluated, and the best solution is updated for the next iteration.

Overall, the original SFOA combines a dimension-sensitive exploration strategy with preying- and regeneration-based exploitation mechanisms. This structure provides a balanced search framework by integrating population diversity, guided movement toward promising regions, and an additional regeneration mechanism. Therefore, SFOA constitutes a suitable baseline optimizer for the hybrid differential evolution-based framework proposed in this study.

### 3.2. Differential Evolution

Differential evolution (DE) is a population-based evolutionary optimization algorithm originally developed for solving continuous global optimization problems [27]. Due to its simple structure, limited number of control parameters, and effective search capability, DE has been widely used in engineering optimization problems. The basic DE procedure is mainly composed of three operators: mutation, crossover, and selection. These operators generate a trial solution from the existing population and decide whether this new solution should replace the current target vector.

Let $X_{i}^{t}=\left[x_{i,1}^{t},x_{i,2}^{t},\ldots ,x_{i,D}^{t}\right]$ denote the i-th target vector at iteration t, where D is the number of decision variables. In the mutation stage, three mutually different individuals, $X_{r_{1}}^{t}$, $X_{r_{2}}^{t}$, and $X_{r_{3}}^{t}$ are randomly selected from the current population, where $r_{1}\neq r_{2}\neq r_{3}\neq i$. The mutant vector $V_{i}^{t}$ is then generated as follows:

(7) $$V_{i}^{t}=X_{r_{1}}^{t}+F\cdot \left(X_{r_{2}}^{t}-X_{r_{3}}^{t}\right)$$

where F∈(0,2) is the mutation scaling factor that controls the amplification of the differential variation between two randomly selected individuals. This mutation strategy allows DE to create a new search direction by using the relative difference between population members.

After mutation, the crossover operator is applied to generate the trial vector $U_{i}^{t}=\left[u_{i,1}^{t},u_{i,2}^{t},\ldots ,u_{i,D}^{t}\right]$. In the commonly used binomial crossover scheme, each component of the trial vector is selected either from the mutant vector or from the target vector according to the crossover rate CR. This operation is defined as:

(8) $$u_{i,j}^{t}=\left\{\begin{array}{c} v_{i,j}^{t}\;\;\;\mathrm{if}\;{rand}_{j}\leq CR\;or\;j=j_{rand}\\ x_{i,j}^{t},\;\;otherwise\;\;\;\;\;\;\;\;\;\;\;\;\;\; \end{array}\right.$$

where $CR\in \left[0,1\right]$ is the crossover rate, ${rand}_{j}$ is a uniformly distributed random number in $\left[0,1\right]$, and $j_{rand}$ is a randomly selected dimension index. The use of $j_{rand}$ ensures that at least one component of the trial vector is inherited from the mutant vector.

In the final stage, a greedy selection mechanism is used to determine whether the target vector or the trial vector survives to the next iteration. For a minimization problem, the selection process is expressed as:

(9) $$X_{i}^{t+1}=\left\{\begin{array}{c} U_{i}^{t}\;\mathrm{if}\;f(U_{i}^{t})\leq f(X_{i}^{t})\\ X_{i}^{t}\;otherwise\;\;\;\;\;\; \end{array}\right.$$

where $f\left(\cdot \right)$ denotes the objective function. This selection rule prevents deterioration in the objective function value and allows the population to gradually move toward better regions of the search space. In this study, DE is not employed as an independent optimizer. Instead, its mutation, crossover, and selection operators are incorporated into the SFOA framework to support population diversity and improve the refinement capability of the proposed hybrid search process.

### 3.3. Hybridization of SFOA and DE

The proposed hSFOA-DE algorithm integrates DE-based mutation, crossover, and selection operators into the exploitation stage of the original SFOA to improve the refinement capability of the search process. In this hybrid structure, the exploration phase of SFOA, defined by Equations (3) and (4), remains unchanged to maintain the original global search capability of the algorithm. Thus, the five-dimensional and unidimensional exploration strategies of SFOA continue to contribute to population diversity during the search process. According to the original SFOA switching mechanism, the exploration phase is performed when $rand>G_{P}$, whereas the exploitation phase is activated otherwise. In the proposed hSFOA-DE structure, the DE-based refinement step is embedded into this exploitation branch. The hybrid exploitation phase is described as follows.

In the DE-based mutation stage, a best-guided mutation strategy is employed to generate a mutant vector around the current promising region. Unlike the classical DE/rand/1 mutation strategy given in Equation (7), the current global best solution is used as the base vector in this hybrid structure. The mutant vector is generated as:

(10) $$V_{i}^{t}=X_{best}^{t}+F\cdot \left(X_{r_{1}}^{t}-X_{r_{2}}^{t}\right)$$

where $X_{best}^{t}$ denotes the current best solution at iteration t, $X_{r_{1}}^{t}$ and $X_{r_{2}}^{t}$ are two randomly selected and mutually different individuals from the population, and F is the mutation scaling factor. This structure provides an exploitation tendency through $X_{best}^{t}$, while the differential term $\left(X_{r_{1}}^{t}-X_{r_{2}}^{t}\right)$ introduces population-based variation into the search process.

After mutation, the binomial crossover operator defined in Equation (8) is applied to generate the trial vector $U_{i}^{t}$. This operation combines the mutant vector $V_{i}^{t}$ with the corresponding target vector and ensures that at least one component is inherited from the mutant solution. Then, the greedy selection rule in Equation (9) is used to determine whether the trial vector should replace the current target vector. Therefore, the DE mechanism is not used as an independent optimizer in the proposed framework; instead, it acts as an additional refinement operator within the exploitation stage of SFOA.

After the DE-based refinement step, the updated candidate solutions are checked against the predefined lower and upper bounds. The regeneration mechanism of SFOA is also retained in the hybrid structure and applied as described in Equation (6). In this way, the proposed hSFOA-DE framework preserves the main search logic of SFOA, including exploration, preying, and regeneration, while incorporating DE-based mutation, crossover, and selection operators to enhance the refinement of promising candidate regions.

From an optimization perspective, the proposed hybridization can also be viewed as a DE-assisted SFOA framework, in which explicit DE mutation and crossover operators are embedded into the SFOA search process. Although SFOA is not identical to DE, both methods belong to population-based search paradigms and may share certain difference-based update characteristics. Therefore, the DE component is introduced here to strengthen candidate variation and local refinement while preserving the exploration, preying, and regeneration mechanisms of SFO.

The main advantage of the proposed hSFOA-DE framework is that it keeps the original search philosophy of SFOA while adding a DE-based refinement operator around promising regions. In the present controller tuning problem, this hybrid structure is expected to improve the search for controller parameters that jointly reduce settling time, overshoot, and accumulated error.

**Figure 1:**
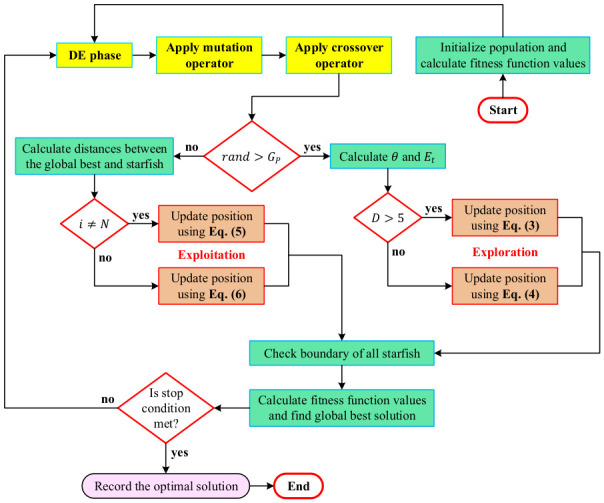
Working mechanism of proposed hSFOA-DE optimizer.

Nevertheless, some limitations should also be acknowledged. The hybrid structure introduces additional algorithmic operations compared to the original SFOA, potentially increasing computational cost. Moreover, its performance may still depend on algorithmic settings such as population size, maximum iteration number, mutation factor, and crossover probability. Therefore, although the proposed hSFOA-DE is designed to improve tuning performance for the considered fan–motor pressure control problem, further validation on different control systems and operating conditions would be useful to assess its broader generalization capability. In line with the structure explained so far, Figure 1 presents the working mechanism of the proposed hSFOA-DE optimizer in detail.

## 4. Dynamic Modelling of the Fan–Motor System

The nonlinear description of a centrifugal fan driven by a squirrel cage three-phase induction motor, experimentally validated in laboratory studies [8,40], is adopted as the system model in this work. This integrated formulation establishes a dependable reference for both controller tuning and simulation studies. The airflow inside the volute is expressed by the following differential relation:

(11) $$\zeta \cdot \frac{dQ}{dt}=H_{r}\cdot {\left(\frac{\omega }{\omega_{r}}\right)}^{2}-\kappa_{1}\cdot Q^{2}-\kappa_{2}\cdot Q-\kappa_{3}\cdot Q^{2}$$

where Q denotes the airflow rate, ω is the fan angular speed, and $\omega_{r}$ stands for its rated value. The constant $H_{r}$ represents the shut-off pressure at nominal speed. Parameters $\kappa_{1}$ and $\kappa_{2}$ approximate the nonlinear flow pressure law, while $\kappa_{3}$ characterizes duct resistance. The coefficient ζ reflects the inertia of the air column in the casing. The instantaneous pressure generated by the impeller is given by:

(12) $$H=H_{r}\cdot {\left(\frac{\omega }{\omega_{r}}\right)}^{2}-\kappa_{1}\cdot Q^{2}-\kappa_{2}\cdot Q$$

The total efficiency of the fan–motor set ($\eta_{tot}$) is defined as the useful hydraulic power divided ($P_{out}$) by the motor’s active input ($P_{in}$):

(13) $$\eta_{tot}=\frac{P_{out}}{P_{in}}=\frac{Q\cdot H}{P_{in}}$$

To overcome the limitations of oversimplified analytical loss models and better represent the nonlinear characteristics of the fan–motor system, a neural network estimator is employed. It is trained based on experimental input–output data, mapping the Q, H, and motor supply frequency f to estimated total system efficiency.

(14) $${\hat{\eta }}_{tot}=ANN\left(Q,H,f\right)$$

where ${\hat{\eta }}_{tot}$ is the ANN estimated total efficiency of the fan–motor system. Accordingly, the fan-only efficiency ($\eta_{fan}$) is derived as:

(15) $$\eta_{fan}=\frac{{\hat{\eta }}_{tot}}{\eta_{motor}}$$

where $\eta_{motor}$ is motor efficiency. $\eta_{motor}$ itself is expressed as the ratio between the developed shaft power (the product of torque T and angular velocity ω) and the sum of shaft power T·ω and power losses $P_{pl}$:

(16) $$\eta_{motor}=\frac{T\cdot \omega }{T\cdot \omega +P_{pl}}$$

where only stator and rotor copper losses are included in $P_{pl}$, while other losses are allocated to the fan side for simplicity. Accordingly, the load torque at the motor shaft is:

(17) $$T_{load}=\frac{P_{out}}{\eta_{fan}\cdot \omega }=\frac{Q\cdot H{\cdot \eta }_{motor}}{\omega \cdot {\hat{\eta }}_{tot}}$$

**Figure 2:**
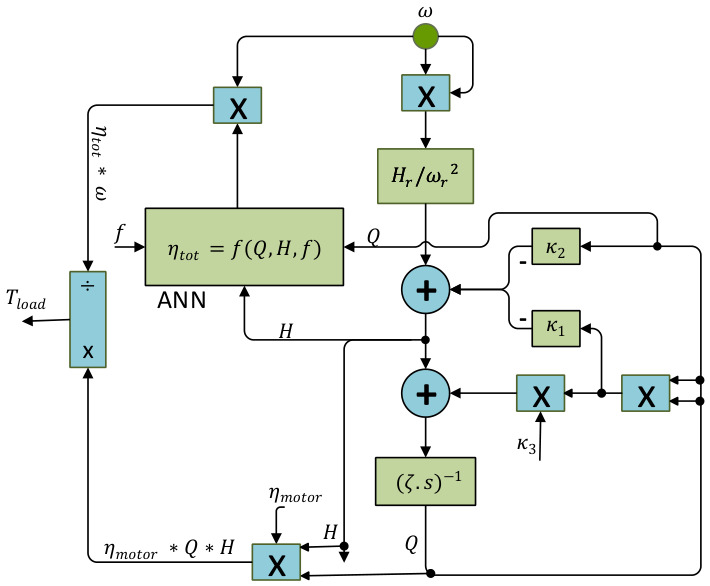
Block diagram of the centrifugal fan.

This relation in Equation (17) links torque demand directly to airflow conditions and efficiency characteristics. The block diagram of the centrifugal fan is shown in Figure 2.

For the induction motor, the conventional well-known five-equation model in the α-β reference frame under scalar control $V/f^{2}$ is utilized. The block implementation in MATLAB/Simulink (2024b) computes angular velocity and currents from input voltages and load torque, also providing copper loss estimates. The integration of the nonlinear fan dynamics with the motor representation yields a combined system consistent with the experimental validation reported in [40], providing a high-fidelity simulation platform, ensuring that the controllers designed on this basis retain practical credibility.

## 5. Proposed Softsign-PI Controller

To overcome the difficulties posed by nonlinear system dynamics and actuator saturation, a novel controller architecture is proposed in which the error signal is first processed by a Softsign function before entering a conventional PI structure. The Softsign function, defined as:

(18) $$softsign\left(x\right)=\frac{x}{1+\left|x\right|}$$

Equation (18) is a smooth nonlinear mapping that compresses large inputs while remaining continuous and differentiable. In the present configuration, the error e(t) is first scaled by a factor $G_{1}$:

(19) $$k\left(t\right)=G_{1}e\left(t\right)$$

and subsequently transformed through the Softsign block with an additional scaling factor $G_{2}$:

(20) $$l\left(t\right)=G_{2}softsign\left(k\left(t\right)\right)=G_{2}softsign\left(G_{1}e\left(t\right)\right)=\frac{G_{1}G_{2}e\left(t\right)}{1+\left|G_{1}e\left(t\right)\right|}$$

**Figure 3:**
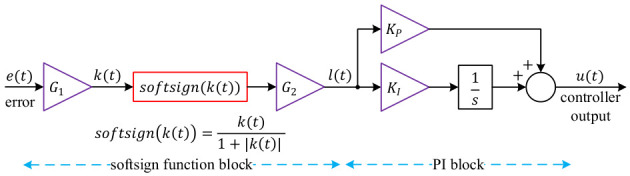
Block diagram of the proposed Softsign-PI controller.

The output of this nonlinear pre-processing stage then drives the PI controller:

(21) $$u\left(t\right)=K_{P}l\left(t\right)+K_{I}\int_{0}^{t}l\left(\tau \right)d\tau$$

where $K_{P}$ and $K_{I}$ denote the proportional and integral gains, respectively. The block diagram representation is given in Figure 3.

While both the Softsign-PI controller structure and the hSFOA-DE algorithm introduce novel elements, the primary focus of this study is on improving pressure regulation performance through the proposed control framework. The hybrid optimization method is employed as a supporting tuning strategy to enhance the controller performance within this application context.

The key novelty of this design is the introduction of Softsign as a nonlinear prefilter preceding a PI controller. While this activation has been extensively employed in neural networks [41] and recently in advanced differentiator/backstepping designs [41], its use within a PI-based architecture has not, to the best of the authors’ knowledge, been reported. Since the Softsign function is inherently bounded within (−1, 1), the pre-processed error signal satisfies$\;\left|l\left(t\right)\right|<G_{2}$ regardless of the magnitude of the original error. By bounding the pre-processed signal within this finite range, the controller is protected from excessively large error inputs, which in practice mitigates the risk of actuator saturation and integrator windup. Unlike hard saturation functions, the Softsign transformation provides a smooth transition that avoids discontinuities liable to compromise stability or excite oscillations. Consequently, this structure offers multiple benefits simultaneously. The bounded nature of $l\left(t\right)$ inherently reduces windup tendencies without requiring auxiliary anti-windup mechanisms. Moreover, the effective controller gain becomes input-dependent: for small tracking errors, the system behaves almost linearly with effective input gain $G_{1}G_{2}$, whereas for large deviations the Softsign block progressively attenuates the action, improving robustness against disturbances and abrupt setpoint changes. From a practical standpoint, this nonlinear pre-processing explicitly addresses actuator constraints by smoothly compressing error magnitudes into a feasible range.

Nevertheless, some limitations should be acknowledged. In scenarios where rapid recovery from large deviations is essential, the bounded input to the PI may slightly slow the transient response compared with an unconstrained PI. Furthermore, the proper selection of the scaling coefficients $G_{1}$ and $G_{2}$ is crucial; inappropriate tuning may degrade responsiveness or compromise tracking precision.

Overall, the proposed Softsign-PI controller preserves steady-state accuracy while introducing saturation-aware, input-dependent shaping, making it a practical enhancement for pressure control in turbomachinery where robustness and actuator protection are essential.

## 6. Objective Function and Application of hSFOA-DE

The tuning of the proposed Softsign-PI controller parameters is formulated as an optimization problem, where the goal is to minimize a multi-criteria fitness function (FF) that reflects both transient and steady-state performance. The weighting coefficients are selected to achieve a balanced trade-off between transient response speed and overshoot, which are critical performance indicators in pressure control applications. Equal weighting ($\rho_{1}=\rho_{2}=0.4$), is adopted to avoid biassing the optimization process toward a single performance criterion, while the remaining term ensures that steady-state accuracy is also taken into account through the IAE component. The fitness function is defined as:

(22) $$FF=\rho_{1}t_{set}+\rho_{2}O_{shoot}+\left(1-\rho_{1}-\rho_{2}\right)\int_{0}^{t_{sim}}\left|e\left(t\right)\right|dt$$

where $t_{set}$ denotes the normalized settling time within a ±2% tolerance band, $O_{shoot}$ is the normalized percent overshoot, and $e\left(t\right)$ represents the instantaneous error between the reference signal and the system output. The simulation time is fixed at $t_{sim}=20\;s$, which provides a sufficiently long horizon to capture the dynamic response characteristics. The selected simulation horizon is sufficient to capture both transient and steady-state dynamics of the system, including settling behaviour and residual steady-state error. If the closed-loop response fails to settle or becomes unstable, the run is penalized by assigning a large FF value.

The decision variables subject to optimization are the Softsign-PI controller parameters ($K_{P}$,$K_{I},\;G_{1}$,$G_{2}$). Their search boundaries are summarized in Table 1. These limits are chosen to avoid impractically large gains that may cause instability, while ensuring that the optimizer has adequate freedom to explore a wide range of feasible solutions.

The hybrid starfish optimization algorithm with differential evolution (hSFOA-DE), described in Section 3, is applied to minimize the above-defined fitness function. During each iteration, candidate solutions for ($K_{P}$,$K_{I},\;G_{1}$,$G_{2}$) are generated, evaluated through the simulation model, and updated according to the hSFOA-DE exploration–exploitation strategies. The optimization process continues until either the maximum iteration count is reached, or satisfactory convergence of the fitness function is obtained.

## 7. Comparative Results and Discussion

### 7.1. Comparison Among Metaheuristic Optimizers

The performance of the proposed hSFOA-DE is benchmarked against SFOA, AOO, EEFO, DE, and PSO. To ensure a fair and reproducible assessment, all algorithms were executed under an identical protocol comprising 25 independent runs, a population size of 30, and 100 maximum iterations. The population size and maximum iteration number were selected to provide an equal computational budget for all compared algorithms. Since the controller tuning problem involves four decision variables, the selected population size of 30 lies within the commonly adopted range for population-based optimizers such as DE, where population sizes are often chosen proportional to the problem dimension. The control parameters of DE and PSO were also assigned based on commonly used settings reported in the literature, and the same stopping criteria were applied to all algorithms to ensure a fair and unbiased comparison.

**Figure 4:**
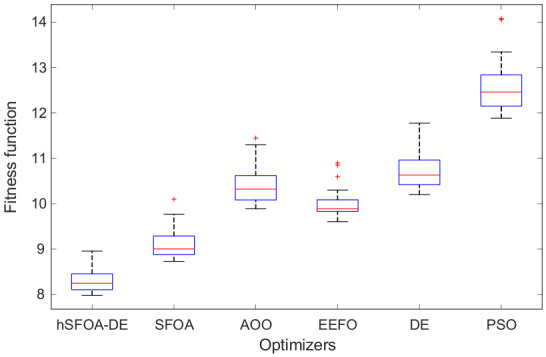
Boxplot analysis of the hSFOA-DE, SFOA, AOO, EEFO, DE and PSO.

As shown in Figure 4, the proposed hSFOA-DE method achieves the lowest median fitness value among all considered algorithms, indicating superior overall solution quality across multiple runs. In addition, the interquartile range (IQR) of hSFOA-DE is relatively narrow, suggesting a consistent optimization performance. Compared with the baseline algorithms, hSFOA-DE exhibits both lower central tendency and controlled variability, although occasional outliers can still be observed due to the stochastic nature of metaheuristic optimization. Other methods, such as AOO, DE, and PSO, show higher median values and, in some cases, wider spreads, indicating less favourable and less consistent performance. Overall, the boxplot results confirm that hSFOA-DE provides a better balance between solution quality and stability across repeated runs.

As summarized in Table 2, hSFOA-DE achieves the best average fitness (8.3123) with the smallest run-to-run standard deviation (0.2568), indicating accuracy and stability. It also attains the lowest minimum (7.9808) and the narrowest min–max span (0.9745), whereas the alternatives exhibit larger spreads. The mean performance rankings further corroborate this ordering: when the six algorithms are sorted by their average fitness across 25 runs, hSFOA-DE ranks first, followed by SFOA (2nd), EEFO (3rd), AOO (4th), DE (5th), and PSO (6th). It should be noted that consecutive ordinal positions are separated by one rank unit by definition, and the actual magnitude of performance differences between algorithms—which varies considerably across pairs—is fully reflected in average and standard deviation rows of the same table, as well as in the distributional spread observable in Figure 4. Taken together, these descriptive statistics confirm the superior solution quality and run-to-run consistency of hSFOA-DE relative to all compared algorithms.

To complement the solution quality statistics, the average CPU time per run was recorded for all algorithms under the same experimental conditions, and the results are reported in the final row of Table 2. AOO exhibits the shortest average runtime (88.3867 s), followed closely by SFOA (90.8336 s) and hSFOA-DE (94.5624 s). EEFO (97.3799 s), PSO (100.8439 s), and DE (105.7380 s) incur progressively higher computational costs. The hybridisation of SFOA with DE mutation and crossover operators introduces an overhead of approximately 3.73 s relative to the base SFOA, corresponding to a marginal increase of roughly 4% in average runtime. Despite this, hSFOA-DE remains faster than three of the five competing algorithms, including both standalone DE and PSO. Considered alongside the substantial improvements in average fitness, run-to-run consistency, and time-domain performance demonstrated throughout this section, these timing results indicate that the performance gains of hSFOA-DE are achieved at an acceptable and proportionate computational cost, supporting the practical viability of the proposed hybrid tuning framework.

**Figure 5:**
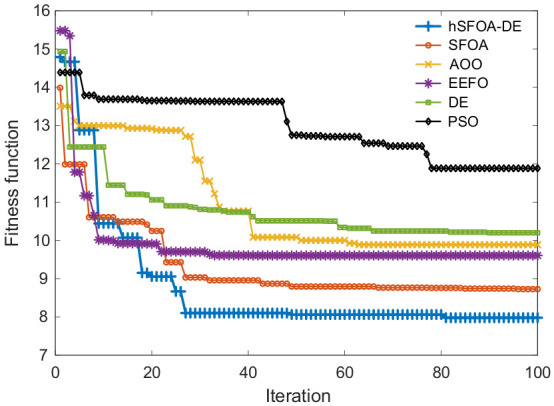
Evolution of fitness function.

As seen in Figure 5, hSFOA-DE drops to ≈8 by about the 25th iteration and then makes small refinements to reach the lowest fitness at iteration 100. SFOA shows a rapid decrease only in the first 3–4 iterations (to ≈12) and thereafter decreases gradually, converging above hSFOA-DE. The lowest final fitness indicates that hSFOA-DE achieves the best overall trade-off among settling time, overshoot, and IAE as defined in Equation (22). Table 3. lists the resulting controller settings—$K_{P}$, $K_{I}$, $G_{1}$ and $G_{2}$—identified by each optimizer.

**Figure 6:**
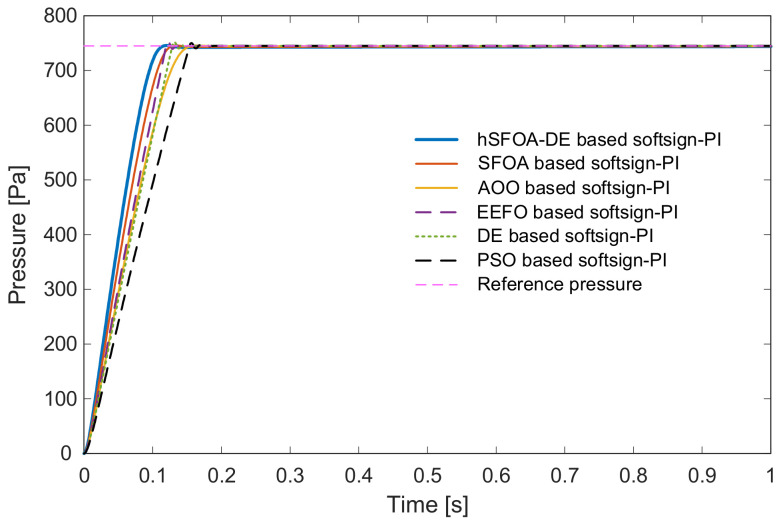
Closed-loop pressure step responses with Softsign-PI (tuned by hSFOA-DE and other algorithms).

**Figure 7:**
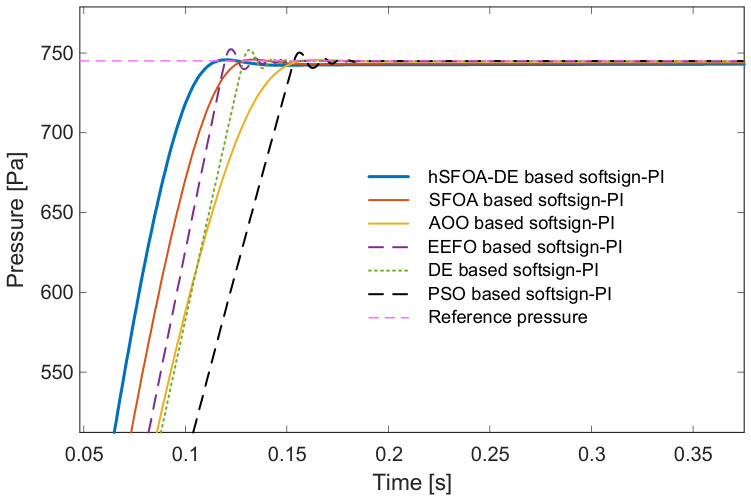
Zoomed view near the setpoint (pressure tracking).

The hSFOA-DE-tuned Softsign-PI reaches the setpoint faster and with a smoother approach than other algorithms showed in Figure 6. The zoomed view in Figure 7 highlights the small transient excursion around the setpoint and the quick return to the tolerance band, evidencing better damping and tracking quality.

As shown in Table 4, where $t_{rise}$ [s] denotes the 10–90% rise time, $M_{p}$ is the peak response and $t_{peak}$ is the time-to-peak. The quantitative indices show that hSFOA-DE achieves the smallest $t_{rise}$, $t_{set}$, $O_{shoot}$, and $t_{peak}$. Moreover, its peak $M_{p}$ is the closest to the setpoint (745 Pa). Taken together, these metrics indicate superior performance and robustness of hSFOA-DE relative to other algorithms.

### 7.2. Comparisons with Classical Tuning Methods

To benchmark against established practice, three classical designs are included: Murrill-based 2-DOF PID, Tyreus–Luyben PID, and Ziegler–Nichols PI. Let $R\left(s\right)$ denote the reference input, Y(s) the plant output, and $E\left(s\right)=R\left(s\right)-Y(s)$ the tracking error in the Laplace domain. The PI controller in the Laplace (s) domain can be expressed as:

(23) $$U\left(s\right)=E\left(s\right)\left(K_{P}+\frac{K_{I}}{s}\right)$$

The PID controller in the Laplace (s) domain can be expressed as:

(24) $$U\left(s\right)=E\left(s\right)\left(K_{P}+\frac{K_{I}}{s}+K_{D}s\right)$$

The 2-DOF PID controller in the Laplace (s) domain can be expressed as:

(25) $$U\left(s\right)=K_{P}\left[\alpha R\left(s\right)-Y\left(s\right)\right]+\frac{K_{I}}{s}E\left(s\right)+K_{D}\frac{Ns}{s+N}\left[\beta R\left(s\right)-Y\left(s\right)\right]$$

where α and β are setpoint weights on the proportional and derivative channels and N is the first-order derivative filter coefficient. All designs are evaluated on the same plant and step reference (745 Pa) used in Section 5. Table 5 lists the tuned parameters produced by each rule.

**Figure 8:**
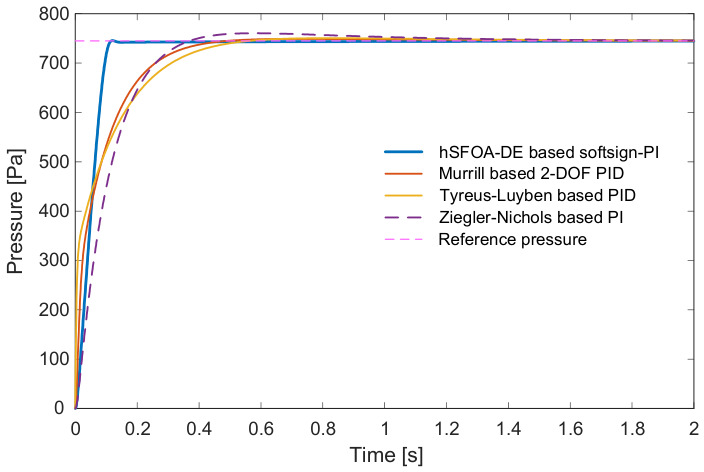
Closed-loop response of hSFOA-DE-based Softsign-PI compared with the classical methods.

As seen in Figure 8, the hSFOA-DE-tuned Softsign-PI reaches approximately 745 Pa setpoint much faster and with a smaller transient excursion than the classical designs (Murrill 2-DOF PID, Tyreus–Luyben PID, Ziegler–Nichols PI). As shown in Table 6, hSFOA-DE achieves the smallest rise and settling times ($t_{rise}=0.0757\;s,\;t_{set}=0.1042\;s$), the lowest overshoot ($O_{shoot}=0.0925\%$), and the earliest peak time ($t_{peak}=0.12\;s$). Its peak value $M_{p}=745.6889\;$Pa is also closest to the 745 Pa reference. In comparison, the classical methods are slower and more oscillatory. Taken together, Figure 8 and Table 6 indicate superior performance and robustness of the hSFOA-DE-tuned Softsign-PI relative to the classical tunings.

### 7.3. Objective Function Results: ZLG and IAE

In addition to time-domain plots, two objective functions, namely ZLG and IAE, are compared across all methods, including both classical tuning approaches and metaheuristic algorithms. ZLG is a composite time-domain index that condenses overshoot, steady-state error, rise time, and settling time into a single score; therefore, it is useful for ranking controllers in terms of overall transient quality and robustness [42]. IAE, on the other hand, measures the accumulated absolute tracking error over the simulation horizon and indicates how effectively the output follows the reference signal.

**Figure 9:**
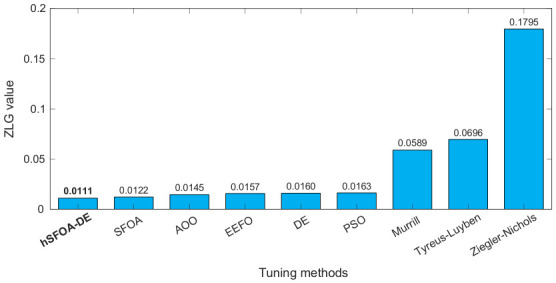
Comparison of ZLG performance indicator values.

As shown in Figure 9, the hSFOA-DE-tuned Softsign-PI controller achieves the lowest ZLG value of 0.0111 among all considered methods. This result indicates that the proposed controller provides the most favourable combined behaviour in terms of overshoot, rise time, settling time, and steady-state error. The metaheuristic-based Softsign-PI controllers generally produce lower ZLG values than the classical tuning methods, which confirms the advantage of optimization-based tuning for this nonlinear fan–motor system. However, among all metaheuristic methods, hSFOA-DE gives the best ZLG value, showing that the hybrid tuning strategy improves the overall transient quality of the closed-loop response.

**Figure 10:**
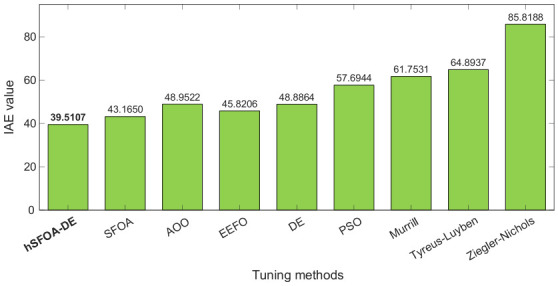
Comparison of IAE performance indicator values.

Figure 10 presents the IAE comparison for the same set of methods. The proposed hSFOA-DE-tuned Softsign-PI controller again yields the lowest IAE value of 39.5107, indicating the smallest accumulated tracking error during the simulation interval. This means that the proposed controller not only reaches the reference rapidly but also maintains smaller deviations from the desired pressure throughout the response. Compared with the classical tuning methods, the reduction in IAE is more pronounced, while the improvement over the other metaheuristic algorithms confirms the additional benefit of the hSFOA-DE search mechanism.

Overall, the results in Figure 9 and Figure 10 demonstrate that the proposed method achieves consistent superiority in both composite dynamic performance and cumulative tracking accuracy. The simultaneous reduction in ZLG and IAE confirms that the improved response is not limited to a single metric, but reflects a balanced enhancement in transient behaviour, steady-state accuracy, and robustness.

## 8. Conclusions and Future Work

This work presents a novel Softsign-PI controller tuned by a hybrid metaheuristic algorithm (hSFOA-DE) for pressure regulation in an induction-motor-driven centrifugal fan. On a literature-validated nonlinear fan–motor model, the proposed Softsign-PI + hSFOA-DE design consistently outperforms contemporary metaheuristics (SFOA, AOO, EEFO, DE, and PSO) and classical tunings (Murrill 2-DOF PID, Tyreus–Luyben PID, and Ziegler–Nichols PI). The evidence is consistent across analyses: statistical summaries and boxplots show stronger central tendency with lower run-to-run variability, fitness–evolution curves indicate improved convergence behaviour, and time-domain performance metrics confirm smoother and faster closed-loop response. Moreover, objective function results yield the lowest ZLG and IAE values. Collectively, these findings demonstrate the effectiveness and robustness of the proposed controller–tuner framework for turbomachinery pressure control. The observed reductions in overshoot, settling time, and cumulative error suggest potential improvements in energy efficiency and actuator usage in practical applications. The main findings can be summarized as follows:

• The Softsign-PI structure improves transient response while maintaining steady-state accuracy.
• The hSFOA-DE algorithm enhances parameter tuning performance in terms of convergence and solution quality.
• The proposed method outperforms both classical and metaheuristic-based approaches across multiple performance metrics.
• The combined improvement in ZLG and IAE indicates balanced enhancement in dynamic response and tracking accuracy.

Future work includes hardware validation on fan/pump rigs; evaluation under disturbances and noise; multi-objective formulations; online or adaptive tuning of the Softsign-PI structure; and extensions to PID or 2-DOF control architectures.

## Figures and Tables

**Table 1:** Lower and upper limits of Softsign-PI controller parameters.

Controller Parameter	Lower Limit	Upper Limit
$K_{P}$	0.1	20
$K_{I}$	0.1	20
$G_{1}$	0.01	15
$G_{2}$	0.01	15

**Table 2:** Statistical results obtained across different algorithms.

Statistical Metric	hSFOA-DE	SFOA	AOO	EEFO	DE	PSO
Minimum	7.9808	8.7277	9.8874	9.6054	10.1992	11.8839
Maximum	8.9553	10.0988	11.4476	10.8973	11.7729	14.0824
Average	8.3123	9.1232	10.4039	10.0063	10.8004	12.6101
Standard deviation	0.2568	0.3506	0.4150	0.3396	0.4976	0.5995
Mean performance ranking	1	2	4	3	5	6
Avg. CPU time (s)	94.5624	90.8336	88.3867	97.3799	105.7380	100.8439

**Table 3:** Obtained controller parameters via different algorithms.

Controller Parameter	hSFOA-DE	SFOA	AOO	EEFO	DE	PSO
$K_{P}$	18.7054	17.1648	14.2077	16.4319	15.4546	18.8889
$K_{I}$	19.3335	19.7197	19.9693	0.1046	2.2621	0.5902
$G_{1}$	0.01097	0.01251	0.01080	8.5573	7.6725	9.2632
$G_{2}$	10.1339	9.4923	9.8113	7.8867	7.7508	5.3979

**Table 4:** Time-domain performance and robustness metrics for metaheuristic methods.

Stability Metric	hSFOA-DE	SFOA	AOO	EEFO	DE	PSO
$t_{rise}$ [s]	0.0757	0.0854	0.1018	0.0912	0.0984	0.1182
$t_{set}$ [s]	0.1042	0.1165	0.1393	0.1170	0.1258	0.1501
$M_{p}$ [Pa]	745.6889	745.8970	745.7677	752.3469	751.9217	750.3065
$O_{shoot}$ [%]	0.0925	0.1204	0.1030	0.9862	0.9291	0.7123
$t_{peak}$ [s]	0.1200	0.1340	0.1650	0.1220	0.1310	0.1560

**Table 5:** Parameter value of classical tuning methods.

Controller Parameter	Murrill-Based 2-DOF PID	Tyreus–Luyben Based PID	Ziegler–Nichols Based PI
$K_{P}$	0.2318	0.2250	0.1643
$K_{I}$	0.2873	0.3581	0.3135
$K_{D}$	0.007206	0.01319	---
N	98.9510	---	---
α	1.0252	---	---
β	0.9147	---	---

**Table 6:** Time-domain performance and robustness metrics comparison with classical methods.

Stability Metric	hSFOA-DE	Murrill	Tyreus–Luyben	Ziegler–Nichols
$t_{rise}$ [s]	0.0757	0.2026	0.2469	0.2060
$t_{set}$ [s]	0.1042	0.3534	0.4231	0.6579
$M_{p}$ [Pa]	745.6889	749.0670	750.6236	760.6230
$O_{shoot}$ [%]	0.0925	0.5459	0.7548	2.0970
$t_{peak}$ [s]	0.1200	0.7260	0.8240	0.5780

## Data Availability

All produced data are available within the manuscript.
